# β-Cyclodextrin as a Functional Excipient Used for Enhancing the Diminazene Aceturate Bioavailability

**DOI:** 10.3390/pharmaceutics11060295

**Published:** 2019-06-22

**Authors:** Narcisa Marangoci, Daniel Timpu, Andreia Corciova, Cornelia Mircea, Anca-Roxana Petrovici, Alina Nicolescu, Elena-Laura Ursu, Valentin Nastasa, Andra-Cristina Bostanaru, Mihai Mares, Mihaela Pertea, Mariana Pinteala

**Affiliations:** 1 Centre of Advanced Research in Bionanoconjugates and Biopolymers, “Petru Poni’’ Institute of Macromolecular Chemistry, 41A Aleea Grigore Ghica Voda, 700487 Iasi, Romania; nmarangoci@icmpp.ro (N.M.); dtimpu@icmpp.ro (D.T.); alina@icmpp.ro (A.N.); ursu.laura@icmpp.ro (E.-L.U.); 2 “Grigore T. Popa”, University of Medicine and Pharmacy, 16 Strada Universitatii, 700115 Iasi, Romania; acorciova@yahoo.com (A.C.); corneliamircea@yahoo.com (C.M.); pertea_mihaela@yahoo.com (M.P.); 3 Ion Ionescu de la Brad, University of Agricultural Sciences and Veterinary Medicine, 8 Aleea Sadoveanu, 700489 Iasi, Romania; vnastasa67@gmail.com (V.N.); acbostanaru@gmail.com (A.-C.B.); mycomedica@gmail.com (M.M.)

**Keywords:** diminazene aceturate, cyclodextrins inclusion complex, antioxidant, bioavailability

## Abstract

In this study, we proposed formulations of diminazene aceturate (DA) designed to improve its bioavailability and to maximize the therapeutic index in animals by overcoming the rapid degradation under the acidic pH of the stomach. An important consequence is the fact that its amount in the bloodstream is close to the administered dose. This was made possible by incorporating DA into the β-cyclodextrin’s (βCD) cavity in a molar ratio of 1:1. The structure of the resulted inclusion complex was established by Raman, DSC, and Wide-Angle X ray Diffraction (WAXD) in solid state and by ^1^H-NMR and H-H ROESY in aqueous solutions. The stoichiometry of the DA:βCD inclusion complex was obtained by using the continuous variation method (Job’s plot), considering the chemical shifts variations of protons from both DA and βCD compounds in ^1^H-NMR spectra. The biological activity was estimated in vitro by antioxidant activity and in vivo by comparing the bioavailability of parent DA and its inclusion complexes after a single dose administration in Wistar rats by using the HPLC method on their blood plasma. In vitro tests showed an improved antioxidant activity. In vivo tests have shown that the DA concentration is always much higher in blood plasma of rats when DA:βCD inclusion complex of 1:1 molar ratio was administered (i.e., at 60 min, DA is around 11 and 3 times higher when DA:βCD inclusion complex of 1:1 molar ratio was administered than the parent DA one and DA:βCD lyophilized mixture of 1:2 molar ratio, respectively).

## 1. Introduction

In our day, the introduction of more concepts in supramolecular chemistry, which permits the studies of self-assembly of molecules (including polymers), has attracted more and more scientists, becoming a popular area for experimental chemistry, creating a new way for the future not only for the chemists, but for the other researchers (biochemists, engineers, physicists, theoreticians, etc.). Supramolecular chemistry is exploiting physical bonds (like hydrogen bonds, π–π stackings, or coordination complexation), which are responsible for fabricating new supramolecular materials [1].

**Figure 1:**
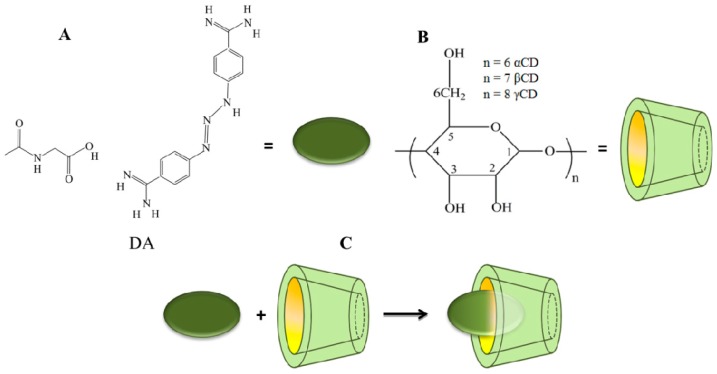
The chemical structures of: (**A**) DA, (**B**) βCD, and (**C**) schematic representation of the inclusion complex formation.

It should be mentioned that an important factor of supramolecular materials is the reversibility of the formed systems and in this respect, inclusion complexation based on cyclodextrins CDs plays a remarkable role. CDs are cyclic oligosaccharide in which several α-d-glucopyranosyl units are connected by 1–3 linkages, forming a truncated cone with a hydrophobic cavity and hydrophilic exterior. α-, β-, and γ-cyclodextrins (6, 7, and 8 d-glucose residue respectively), (Figure 1B) are the most used for the encapsulation of hydrophobic molecules into their cavities, forming host–guest inclusion complexes [2,3,4,5], enabling their use in pharmacology. By using the CDs in different formulations, the solubility, stability, sensory properties, and biological effects of the guest molecules are improved, and also, from the point of view of the technological process, simple handling, standardized compositions, reduced labor cost etc., can be achieved [6,7,8]. 

DA, an aromatic diamidene (4,4′-(1-Triazene-1,3-diyl)bis(benzenecarboximidamide)) (Figure 1A), has attracted attention in recent years due to its therapeutic potential. DA is able to control animal trypanosomiasis and babesiosis [9], being widely used especially in countries of the African continent, Southeast Asia, and South America [10]. Also, new and promising possible pharmacological applications have been found in animal models, such as: asthma [11], gastric mucosal damage [12], ischemic stroke [13], uveitis [14], glaucoma [15], and myocardial infarction [10] by activation of angiotensin-converting enzyme 2 (ACE2). Moreover, it was reported to have the ability to modulate the immune system and thus be able to be used in rheumatoid arthritis by inhibition of the activity of synovial fibroblasts [16]. Other authors showed the involvement in neurodegenerative disorders, such as Huntington’s [17] and Alzheimer diseases [18]. Recently, Ge et al. [19] have shown that DA may have anti-inflammatory activity in cases of inflammatory liver disorders. 

Most studies show that DA has both antioxidant and antimicrobial activity more or less pronounced according to the administered dose. At the same time, an important problem that must be overcome is to prevent the administration of high doses that may be lethal [20]. As for its chemical stability in aqueous solutions, DA is stable for a few days at neutral pH, while in acidic pH, the molecule is transformed in a few minutes in 4-amino-benzamidine and 4-amino-phenyl-diazonium salt [21]. Besides this, it is strongly recommended to protect DA against direct light due to its photo-instability [22]. 

As a consequence, further formulations are necessary for DA to ensure a better bioavailability and to maximize the therapeutic index in various animal species, overcoming the rapid degradation in acidic pH of the stomach and photo-instability, and finally decreasing in its therapeutic doses. Also, it has been mentioned that only simply mixing βCD with DA has an increased therapeutic effect on Ichthyophthyrius multifiliis treatment in farmed carp beetles [23]. 

In this context, by complexing DA into the cavity of a cyclodextrin, a positive impact on the physical (solubility and stability enhancement) and biological properties is expected. 

Taking all of these aspects into consideration with the work done before in which CDs were used to protect the drugs against degradation in the stomach at low pH and, consequently, increasing the drug release rate into blood, in this work we propose the complexation of DA into the βCD cavity (Figure 1C). The structure of the resulting inclusion complex was established by Raman, Proton ^1^H-NMR, H-H ROESY, X-ray Diffraction Spectroscopy, and DSC methods. The complex stoichiometry was obtained from a Job’s plot. The biological activity was estimated in vitro by antioxidant activity and in vivo by comparing the bioavailability of parent DA with its complex with βCD after a single dose administration in Wistar rats by using the HPLC method on their blood plasma.

## 2. Materials and Methods

### 2.1. Materials

All reagents and solvents were purchased from Sigma-Aldrich (Merck KGaA, Darmstadt, Germany) and were used as received.

### 2.2. Solid Inclusion Complex Preparation

Synthesis of the DA:βCD inclusion complex of 1:1 molar ratio was done by co-precipitation, followed by the freeze-drying method [24]. A 1:1 molar ratio of DA to βCD aqueous solution (c_βCD_ = 50 mg/mL) was stirred continuously at room temperature for 48 h, until the solution became slurry. The obtained mixture was immersed in liquid nitrogen and was submitted for freeze-drying for 24 h in a Martin Crist, ALFA 1-2LD freeze-dryer. The raw product was washed under vacuum, with small volumes of cold ultrapure water until the guest and host were not present in the filtrate [25]. The solid inclusion complex was noted as DA:βCD (1:1). 

The preparation of DA:βCD mixture in 1:2 molar ratio was performed in the same manner as the DA:βCD (1:1) complex and was used without purification for in vivo tests. The solid lyophilized mixture was noted as DA:βCD (1:2). 

Physical Mixture Preparation of Diminazene Aceturate (DA) with β-Cyclodextrin (βCD).

Powders DA and βCD, in a molar ratio of 1:1, were mixed in a ceramic mortar prior to analysis.

### 2.3. Stoichiometry of the Inclusion Complex and Association Constant Determination

The stoichiometry of the DA:βCD inclusion complex was determined by plotting the chemical shifts variations of the H-3 protons from βCD (Figure 1B) and aromatic proton from DA (Figure 1A) as a function of the mole fraction of βCD (X_H_) and DA (X_DA_), respectively.

The association constant (Ka) of the DA:βCD inclusion complex was calculated from ^1^H-NMR spectra by applying the NMR version of the Benesi–Hildebrand equation 1/Δ*δ* = 1/Ka Δ*δ*_max_[H]_0_ + 1/Δ*δ*_max_, where, Δ*δ* is the observed chemical shift difference of the guest for a given cyclodextrin concentration ([H]_0_) and Δδ_max_ is the chemical shift difference between a pure complex and the free guest [26].

### 2.4. Physico-Chemical Characterization of DA:βCD Inclusion Complexes

*Raman spectra* were acquired using dried samples with an InVia Raman Microscope (Renishaw, Gloucestershire, UK), equipped with a He–Ne laser at 632.8 nm (17 mW) and a CCD detector coupled to a Leica DM 2500 M microscope. All measurements were performed in backscattering geometry using a 100× objective. The Raman measurements were performed at room temperature and atmospheric pressure. Spectral manipulations were performed with the WiRE 3.2 software (Renishaw, Gloucestershire, UK). 

*DSC curves* were recorded on a DSC 200 F3 Maia device (Netzsch, Selb, Germany). A sample mass of 2.5 mg was heated in aluminum crucibles covered with pierced and pressed lids at a 10 °C min^−1^ heating rate in nitrogen atmosphere at a flow rate of 50 mL min^−1^. Device calibration was made with indium. 

Wide-Angle X ray Diffraction (WAXD), analyses were performed on a Diffractometer D8 ADVANCE (Bruker AXS, Karlsruhe, Germany), using the Cu-Kα radiation (λ = 0.1541 nm), a parallel beam with Gobel mirror and a Dynamic Scintillation detector. All the samples were investigated for a *2θ* diffraction angle ranging from 2° to 50°, in stepwise mode to have more accurate diffraction line shapes (count time 2 s/step, step size 0.02°, at 40 kV and 35 mA), at room temperature. The calculus of *2θ* values, interplanar distance d, and peak heights were carried out with Bruker “DIFFRAC-PLUS Evaluation–EVA” software. Bruker “TOPAZ” software was used for crystallites size data processing. The calculus was performed according the Bragg’s law and Debye-Scherrer formula.

^1^H-NMR spectra have been recorded on a Bruker Advance NEO spectrometer operating at 400.1 MHz for ^1^H, using D_2_O as a solvent. For the NMR analysis, a 5 mm multinuclear direct detection z-gradient probe was used. ^1^H-NMR spectra and H–H ROESY spectrum were recorded using standard pulse sequences, as delivered by Bruker, with TopSpin 4.0.3 spectrometer control and processing software.

### 2.5. DA Calibration Curve 

From a DA stock solution of 5 mg/mL in Milli-Q water, serial dilutions were made (0.05; 0.1; 0.3; 0.5; 0.7; 0.9 mg/mL) to obtain the calibration curve as DA concentration versus pick area of the registered chromatogram.

### 2.6. Stability Studies of DA and DA:βCD (1:1) in Acidic and Neutral pH

Sample preparation: 5 mL aqueous solutions of DA and DA:βCD (1:1) with concentration of 1 mg/mL DA at pH = 1.2 (using HCl) were prepared (each solution contains 5 mg DA). At different time intervals (0, 0.5, 1, 2, 3, 4, and 48 h), 10 µL of each sample were withdrawn and the HPLC chromatograms were recorded (ESI, Figure S2). 

### 2.7. Antioxidant Activity of the DA:βCD (1:1) Inclusion Complex

In order to determine the antioxidant activity, the ability of DA and its inclusion compound was used to inhibit the lipoxygenase activity by a modified Malterud method [27], as previously reported by the authors in Reference [3]. The samples’ concentrations ranged between 0.0781 and 1.25 mg/mL for DA and βCD, and between 0.0234 and 0.375 mg/mL for the DA:βCD inclusion complex. 0.05 mL of the tested samples were treated with a 0.05 mL solution containing lipoxygenase in borate buffer (pH 9) and after 10 min, 2 mL of linoleic acid solution in borate buffer medium was added. The absorbances of the solutions were recorded at 234 nm, and the ability to inhibit the lipoxygenase activity and the IC50 (expressed in μg/mL substance in final solution) were calculated.

### 2.8. Evaluation of the Drug Release Profile in Blood Plasma from Treated Rats 

#### 2.8.1. Animals

A total number of 15 adult female outbred Wistar rats (mean weight 542 ± 37 g) were used (Cantacuzino Institute, Bucharest–Romania). The animals were divided into 3 groups of 5 rats each, hosted in 1500 cm^2^ polycarbonate cages, and let to acclimatize for 48 h until the day of the experiment. During this period, all animals had unrestricted access to standard feed and tap water. The following standard microclimate conditions were assured: temperature 22 ± 0.7 °C, relative humidity 60% ± 10%, and a cycle of 12-h light/12-h dark. Twelve hours before the experiment, the feeding was suspended in order to assure an empty stomach at the moment of gavage.

#### 2.8.2. Experimental Design

DA and the inclusion complexes, DA:βCD molar ratio 1:1 and DA:βCD molar ratio 1:2, were tested. All compounds were solubilized in distilled water and a final concentration of 5 mg/mL DA was assured.

Animals from group A received a single dose of 10 mg/kg DA orally (by gavage), while those from groups B and C received a similar dose of DA, but as a complex with βCD, DA-βCD 1:1 and DA-βCD 1:2, respectively.

After 30, 60, 120, and 180 min respectively, 1 mL blood samples were collected from each animal by cardiac puncture under anesthesia with ketamine 50 mg/kg. The blood was mixed with potassium EDTA to prevent the coagulation, and further processed by centrifugation at 3000 *g* in the first 30 min after the puncture in order to obtain plasma. 

After the experiment, all animals were euthanized using injectable pentobarbital sodium (Euthanyl^®^).

The study fully complies with the recommendations of Directive 2010/63/EU and was approved by the Ethics Committee of the institution through no 7 from 07.01.2019 Animal test approve. 

#### 2.8.3. HPLC Determination of the Diminazene Aceturate (DA) from Rats’ Plasma

The HPLC determination of DA from rats’ plasma was carried out using a Perkin Elmer HPLC system with a Flexar UV/VIS Detector (Waltham, Massachusetts, USA) at 254 nm. A 10 µL fixed injection loop was made in a Zorbax SB-C8 (München, Germany), column (5 µm, 150 mm × 4.6 mm) with a 0.5 mL/min flow mobile phase consisting in methanol-acetonitrile-Milli-Q water (10:10:80 *v*/*v*/*v*), adjusted to pH 4 with 100% formic acid [28].

#### 2.8.4. Blood Samples Preparation

Prior to the HPLC analysis for quantitative diminazene aceturate determination, the sanguine plasma must be purified by deproteinization. One volume of plasma was vortexed for 20 s with half volume of 10% hydrochloric acid. Proteins were separated by centrifugation at 4000 rpm for 10 min and the pH of the supernatant was neutralized at 7 with 4M potassium hydroxide [29]. 

### 2.9. Statistical Analysis

Data were expressed as the mean value ± standard deviation (SD) obtained from three measurements. The statistical analysis was performed using the Student’s *t*-test and the differences at *p* < 0.05 (95% confidence level) were considered to be significant.

## 3. Results and Discussion

The solid DA:βCD inclusion complex of 1:1 molar ratio, obtained by co-precipitation and followed by freeze-drying, was analyzed from the point of view of structure, stoichiometry, and association constant. The biological activity was estimated in vitro (by the determination of the inhibition of lipoxygenase activity) and in vivo (on Wistar rats). For comparison, native DA and DA:βCD inclusion complex of 1:2 molar ratio were used.

### 3.1. Physico-Chemical Characterization of the DA:βCD (1:1) Inclusion Complex

#### 3.1.1. Raman Analysis of the DA:βCD (1:1) Inclusion Complex

**Figure 2:**
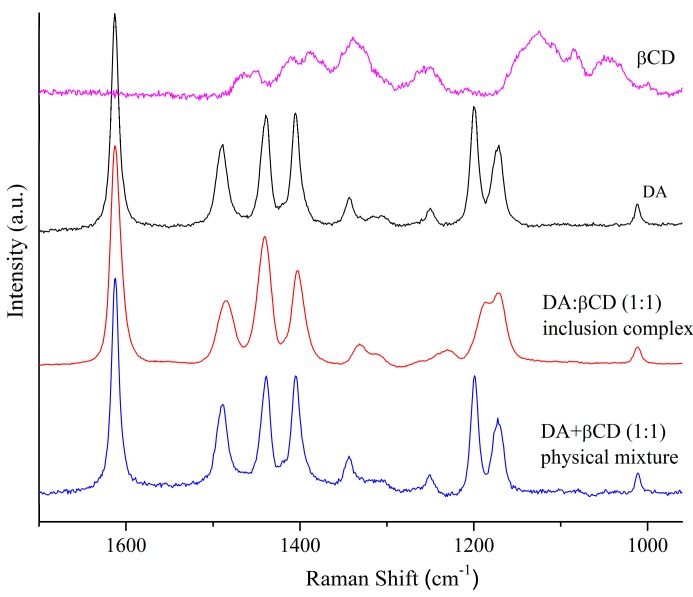
Raman spectra of DA, βCD, and the DA:βCD (1:1) inclusion complex and DA + βCD physical mixture of 1:1 molar ratio in the 1700–950 cm^−1^ wavenumber range.

Figure 2 presents the Raman spectra of dried forms of DA, βCD, and the DA:βCD (1:1) inclusion complex and DA + βCD physical mixture of 1:1 molar ratio in the 1700–950 cm^−1^ wavenumber range. By comparing the Raman spectra of the individual DA and βCD components with their 1:1 physical mixture, approximately the sum of the individual molecules was achieved in the Raman spectrum of the physical mixture. From a close introspection on the DA:βCD inclusion complex spectrum, the main characteristic features of DA and some additional contributions caused by the host–guest interaction can be observed. In this spectrum, a special attention is given to the region above 1550 cm^−1^ where no interfering bands from βCD are present and the changes induced by DA complexation into CD’s cavity can be evidenced [30]. DA in this range exhibits a characteristic peak at 1613 cm^−1^ corresponding to stretching vibrations of the C=N group [31] and after its complexation, a slight shift from 1613 to 1611 cm^−1^, accompanied by its broadening (10.3 cm^−1^ for DA; 13.9 cm^−1^ for DA:βCD) can be detected. The broadening of this signal seems to confirm the existence of weak interactions between the cyclodextrin’s cavity and the DA atoms involved in the mode assigned to this band [32]. The 1400–1500 cm^−1^ band of DA are due to C–H deformation vibrations, C–C stretching vibrations of the phenyl ring, and N=N stretching vibrations, but the exact assignment is very difficult since in this region a mixing of vibrations is possible [33,34]. It should also be noted that the bands from 1409, 1492, and 1435 cm^−1^ of DA are shifted at 1406, 1489, and 1437 cm^−1^ respectively, in the DA:βCD spectrum. Going towards lower wavenumbers, the peaks of DA from 1200 cm^−1^ and 1172 cm^−1^, which are attributed to C–H scissoring of the phenyl ring and C–N stretching vibrations, are shifted to a lower wavenumber in a shoulder with maxima at 1184 and 1170 cm^−1^ respectively, in the DA:βCD (1:1) inclusion complex. Since the positions of the peaks are shifted accompanied with changes in their intensities in the DA:βCD spectrum, as compared with the free DA spectrum, it is possible to conclude that an interaction between DA and βCD has occurred.

#### 3.1.2. DSC Analysis of the DA:βCD (1:1) Inclusion Complex

**Figure 3:**
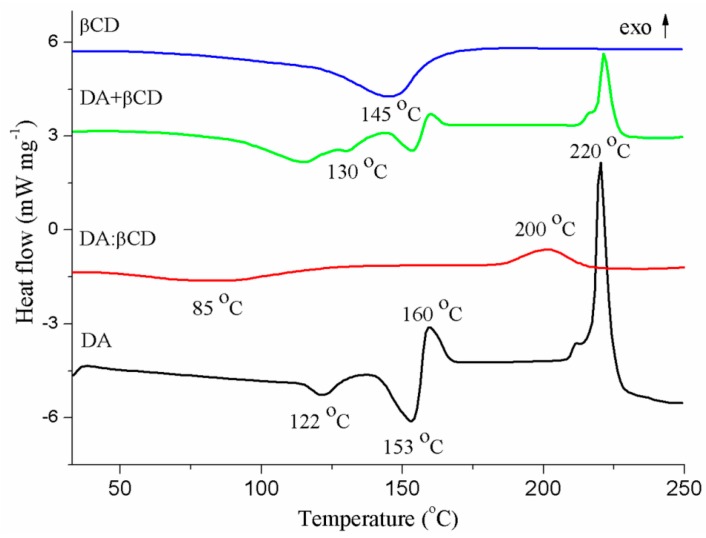
DSC curves of the DA, βCD, and the DA:βCD inclusion complex and DA + βCD (1:1 molar ratio) physical mixture.

Figure 3 shows the DSC curves of the studied structures. DSC is an analytical method used for identifying host–guest solid-state interactions. Through complexation, the guest molecule is protected by the host molecule. This aspect is usually reflected in reduced and/or displaced melting and decomposition profiles of the guest molecule in the inclusion complex [35,36]. The broad endothermic profile (Figure 3) centered at 145 °C corresponds to the loss of crystalized water molecules from the βCD cavity. The DA molecule exhibits two endothermic profiles, at 122 °C and 153 °C, related to salt dehydration. The two endothermic dehydration profiles of the pure drug, together with that of the βCD (130 °C), may also be observed in the DSC curve of the physical mixture. The drug thermally decomposes in two phases, described by the exothermic peaks at 160 °C and 220 °C, which also appear in the DSC curve of the DA in its free state [36]. By analyzing the DSC curve of the DA:βCD inclusion complex, it may be observed that the two thermal decomposition profiles of the drug coalesce into a significantly reduced one, displaced at 200 °C. This is an indication of the loss of the drug’s crystallinity with a new solid phase formation through the complexation of DA inside the βCD cavity [2,37,38].

#### 3.1.3. Wide-Angle X ray Diffraction (WAXD) Analysis of the DA:βCD (1:1) Inclusion Complex 

The βCD and DA precursors are crystalline powders with sharp peaks, which are not influenced by the freeze-drying process or by wetting treatment (ESI, Figures S2 and S3). In both cases, thin shifts of the peak positions can be observed, maybe due to the internal tensions induced by the freeze-drying process. It should be noted that no changes in the initial peaks of βCD (ESI, Figure S4) and DA (ESI, Figure S5) were observed after their storage for 18 h in a saturated water vapor atmosphere.

**Figure 4:**
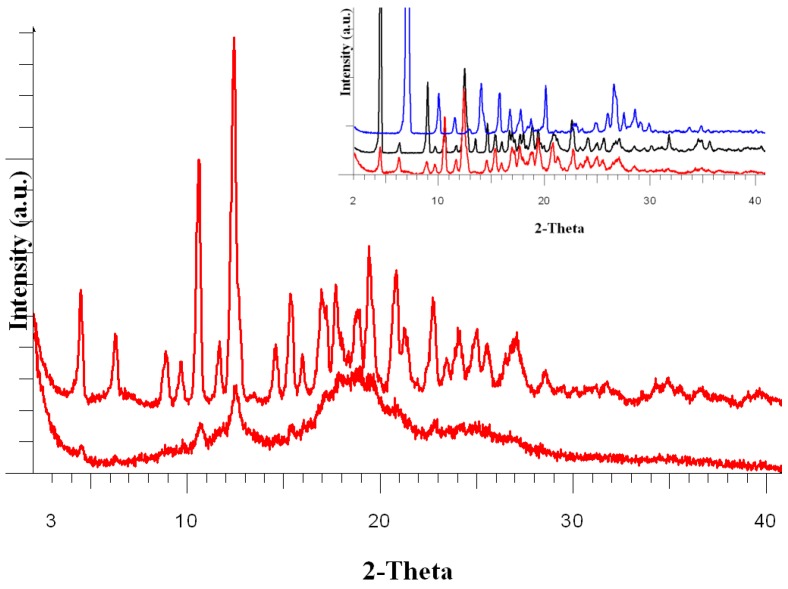
Wide-Angle X ray Diffraction (WAXD) diffractograms of the DA:βCD (1:1) inclusion complex: after the freeze-drying process (red lower diffractogram) and after 18 h maintained in a water saturated atmosphere (red upper diffractogram). Insert: WAXD diffractograms of the DA:βCD (1:1) inclusion complex after 18 h maintained in a water saturated atmosphere (red), βCD peaks (black-middle diffractogram) and DA (insert, blue-upper diffractogram)**.**

After the obtaining of the DA:βCD (1:1) inclusion complex, both DA and βCD precursors lost their crystallinity, (Figure 4, lower diffractogram) and the complex became amorphous as a result of the freeze-drying process; only some small signals remain, perhaps due to the contamination of the complex with traces left of pure βCD.

In order to better understand the structure of the complex, we kept the DA, βCD, and DA:βCD (1:1) inclusion complex in a saturated atmosphere with water vapors for 18 h. After this, the crystallinity of the complex was restored (Figure 4, red upper diffractogram and inserted image). The peaks of the complex with restored crystallinity (Insert Figure 4, red-lower diffractogram) have the same 2-theta positions, like the initial βCD peaks (Insert Figure 4, black-middle diffractogram). No peaks for the initial DA appeared (Insert Figure 4, blue-upper diffractogram), this situation may be explained by the fact that the inclusion of DA into the βCD’s cavity is a “molecule to molecule” process, so the crystalline structure (long-distance order) for DA molecules is impossible to appear. 

The DA:βCD (1:1) complex was also kept for 36 h in the saturated atmosphere with water vapors, no changes appeared in the registered diffractograms (ESI, Figure S4). 

#### 3.1.4. NMR Spectroscopy Analysis of DA:βCD (1:1) Inclusion Complex 

NMR spectroscopy is a well-established technique for the characterization of host–guest interactions. In order to determine the stoichiometry of the DA:βCD complex, 10 mM stock solutions of DA and βCD were prepared in D_2_O. Starting from these stock solutions, a series of nine samples were obtained, containing both DA and βCD, with mole fractions varied from 0 to 1. Different volumes from each stock solution were mixed so that the total concentration of the final solutions was kept constant to 10 mM. The resulting solutions were transferred into 5 mm NMR tubes and the corresponding ^1^H-NMR spectra were recorded. 

**Figure 5:**
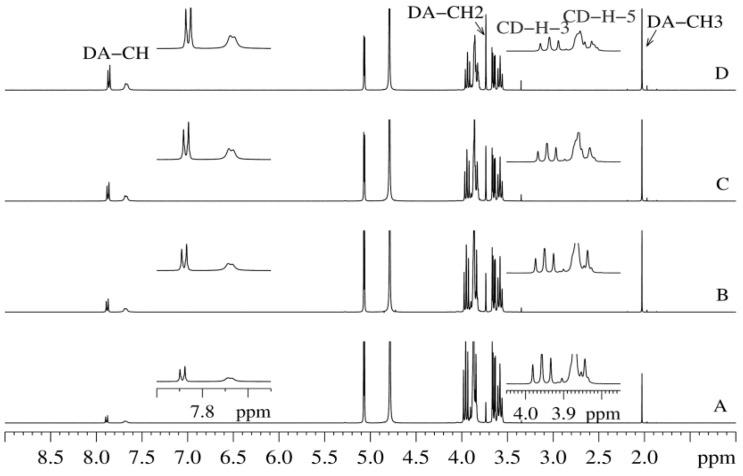
Full ^1^H NMR spectra of DA:βCD mixtures, with mole fractions varied from 0 to 1: (**A**) 0.125 DA and 0.875 βCD; (**B**) 0.250 DA and 0.750 βCD; (**C**) 0.375 DA and 0.625 βCD; (**D**) 0.500 DA and 0.500 βCD. The inserts are showing upfield shifts for DA aromatic protons and H-3 and H-5 βCD protons, with increasing the DA mole fraction.

We followed the DA inclusion into the hydrophobic βCD’s cavity by measuring the variations of some protons’ chemical shifts of both guest and host molecules. Figure 5 presents the obtained ^1^H-NMR spectra for the DA:βCD mixtures, with mole fractions varied from 0 to 1. Changes of the resonance frequencies were observed for the aromatic protons from DA and βCD protons. 

**Figure 6:**
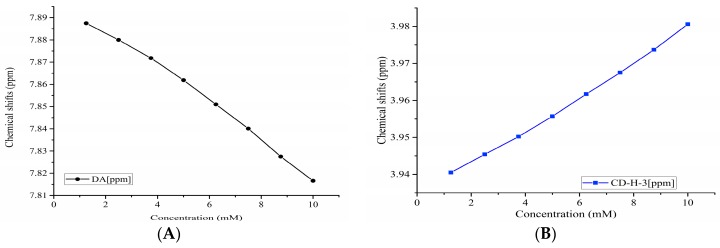
Chemical shifts variation as a function of βCD concentration of: (**A**) 7.82 ppm protons of DA and (**B**) H-3 of βCD protons.

The stoichiometry of the DA:βCD inclusion complex was obtained by using the continuous variation method (Job’s plot), considering the chemical shifts variations of protons from both DA and βCD. One of the two DA’s aromatic signals has the shape of a broad doublet (Figure 5) and as a consequence, its chemical shift variation is more difficult to follow due to its reading errors. Because of this, we chose to follow the signal from 7.82 ppm only, which has the shape of a well-defined doublet. Regarding the βCD protons, we chose to follow the chemical shift variation of the H-3 signal, which is less susceptible to reading errors. The H-5 signal, which is also of interest in complexation NMR studies, is partially overlapped by the H-6 signal, making it more difficult to be observed. The ^1^H chemical shifts variations of these representative protons as a function of the βCD concentration are presented in Figure 6. 

It should be mentioned that the chemical shifts of the protons from aceturate residue were not observed in ^1^H-NMR studies. 

**Figure 7:**
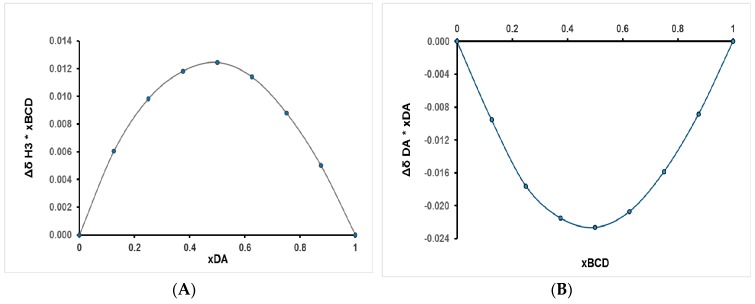
Job’s plots corresponding to chemical shifts variations of: (**A**) H-3 protons of βCD and (**B**) aromatic proton of DA from 7.82 ppm.

The graphical representations of the induced chemical shifts differences (Δδ*X_DA or βCD_) as a function of mole fraction X_DA or βCD_ are presented in Figure 7A,B. The parameter Δ*δ* is calculated as the difference between the chemical-shifts values in the absence and in the presence of the host or the guest. In both representations, the Job’s plot has a maximum at 0.5, indicating the formation of a complex with 1:1 stoichiometry. 

**Figure 8:**
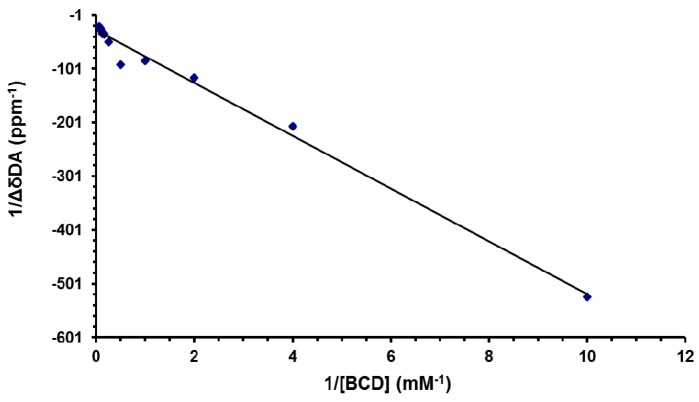
Graphical representation of the Benesi–Hildebrand data treatment.

Ka of the DA:βCD (1:1) inclusion complex was calculated from the ^1^H-NMR data by applying the NMR version of the Benesi–Hildebrand equation (see Section 2.3). In this respect, a new series of thirteen solutions were prepared by keeping the concentration of DA constant at 0.1 mM and varying the βCD concentration from 0.1 to 16 mM. The corresponding ^1^H-NMR spectra were recorded and the chemical shifts variations of the DA signal from 7.82 ppm were noted. In Figure 8, the double-reciprocal plot of the NMR version of the Benesi–Hildebrand equation is represented. 

The straight line confirms the 1:1 stoichiometry of the inclusion complex and from the slope, the value for the Ka was found to be 608.7 M^−1^.

**Figure 9:**
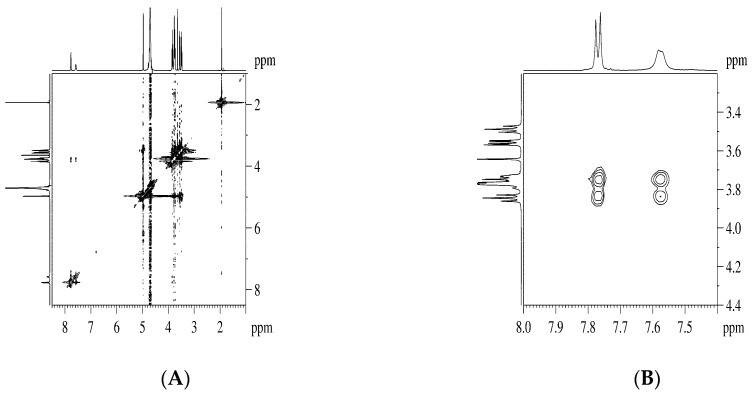
(**A**) The ROESY spectrum of DA:βCD (1:1) inclusion complex, recorded in D_2_O, with suppression of the water signal; (**B**) Expansion of the ROESY spectrum showing the NOE cross-peaks between phenyl protons of DA and internal βCD protons (H-3, H-5).

Supplementary information about the DA:βCD inclusion complex were obtained from the ROESY experiment when a NOE cross-peak is observed if two protons from different compounds are in spatial vicinity within 3–5 Å. The ROESY spectrum corresponding to the 1:1 DA:βCD mixture showed NOE cross-peaks between diminazene aromatic protons and the internal βCD protons H-3, H-5, and H-6 (Figure 9). No NOE cross-peaks were observed between aceturate moiety and βCD. Also, in the case of the DA:βCD lyophilized mixture in a 1:2 molar ratio (named DA:βCD (1:2)), the existence of a diminazene–βCD interaction was demonstrated from the ROESY spectrum, while an aceturate–cyclodextrim interaction has not been confirmed (ESI, Figure S1).

### 3.2. Chemical Stability of DA in Acidic and Neutral pH from DA and DA:βCD (1:1) Aqueous Solutions

**Figure 10:**
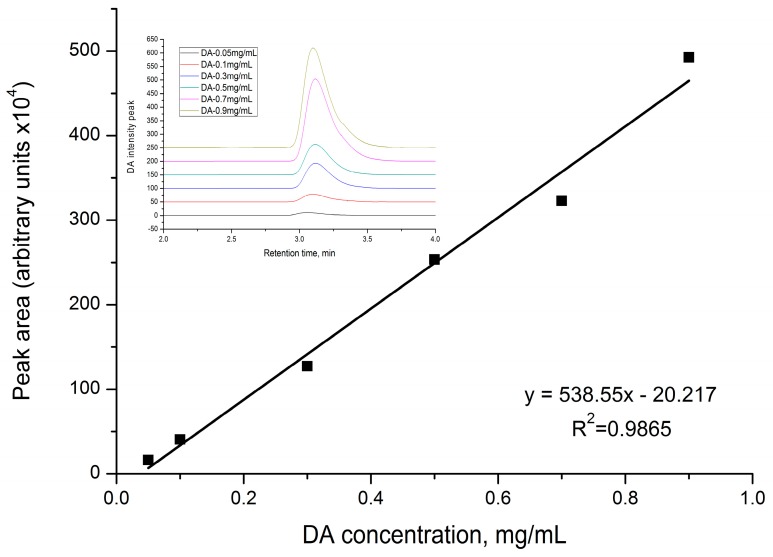
Calibration curve of DA as a function of the DA concentration and peak area of the recorded chromatogram.

**Figure 11:**
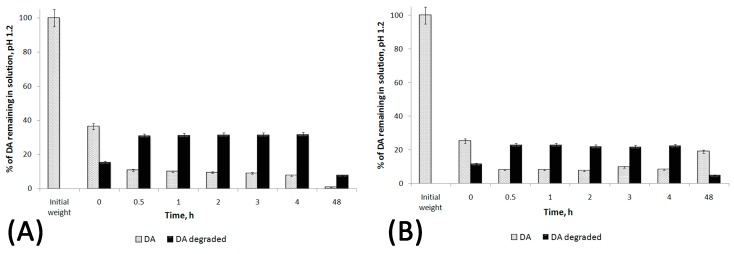
The percentage of DA remaining in water solutions of dissolved DA (**A**) and the DA:βCD (1:1) inclusion complex (**B**) at pH 1.2 and at different time intervals, using HPLC to analyze.

**Figure 12:**
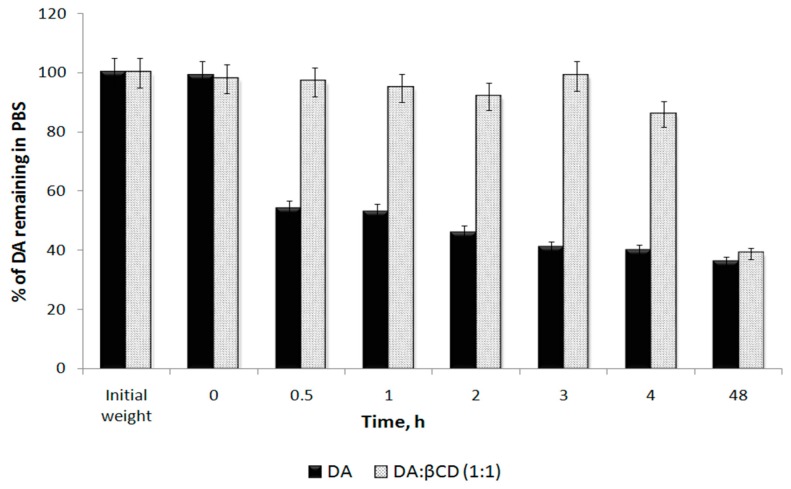
The percentage of DA remaining in PBS solutions of free DA and DA:βCD compounds after HPLC analyses.

We performed the chemical stability at pH = 1.2 and neutral pH of DA in free DA and DA:βCD (1:1) aqueous solutions. At different time intervals (0, 0.5, 1, 2, 3, 4, and 48 h), 10 µL of each sample were withdrawn and the HPLC chromatograms were recorded (ESI, Figures S5 and S6). Using the obtained pick areas of the DA and of the degraded products together with the DA etalon curve (Figure 10), the remaining percentage of DA and percentage of degraded compounds relative to the DA initial amount at different time intervals were calculated (Figure 11 and Figure 12). As it can be observed in ESI, Figure S2, at pH = 1.2, the degradation of DA occurs suddenly, as soon as it is dissolved in solution. All HPLC spectra show two main peaks, one at around 2.3 min and the other one around 2.8 min, being attributed to the diminazene and to the degradation products, respectively. According to Figure 11, it should be noted that the free DA dissolved in water at pH 1.2 is totally degraded in the first 48 h, while the DA from the DA:βCD (1:1) acidic solution is still present in 20% relative to the DA initial amount (5 mg), suggesting that it is a release over time of DA from the cavity of cyclodextrin which acts as a protector to DA. It should also be mentioned that not all existing compounds in the system (e.g., inclusion complex or degraded products) can be detected through the HPLC method [27,39] explaining why not all DA in the system can be detected.

Using HPLC chromatograms of DA and DA:βCD solutions at neutral pH obtained at different time intervals (ESI, Figure S3) and the calibration curve (Figure 10), the percentages of DA remaining in solutions (reported to the initial amount of DA, 5 mg) were calculated (Figure 12). Figure 12 reflects that the DA from both types of solutions (dissolving free DA or the DA:βCD (1:1) inclusion complex) at the initial time is not degraded as in acidic pH (Figure 11), but after 30 min. A degradation process occurred in the free DA solution while in the DA:βCD (1:1) solution the DA is stable for at least 4 h, explaining the ability of ciclodextrin to act as the protector of its guest. After 48 h at room temperature, the DA concentration decreases to less than 50% in both solutions when HPLC chromatograms of DA degradation revealed the presence of a single compound, which indicates that there are no secondary degradation compounds in PBS (ESI, Figure S3).

### 3.3. Biological Activity Determination 

The biological activity of the parent DA and synthesized compounds was estimated in vitro by antioxidant activity, and in vivo by measuring the drug’s concentration in the rat blood plasma at different time intervals after one dose administration, using the HPLC method.

#### 3.3.1. Antioxidant Activity of the DA:βCD (1:1) Inclusion Complex

**Figure 13:**
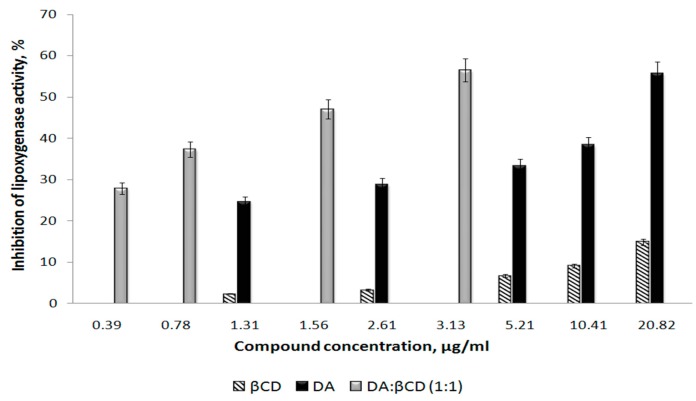
The inhibitory capacity of DA, βCD, and the DA:βCD (1:1 molar ratio) inclusion complex on lipoxygenase activity as a function of DA concentration in the final solution.

Antioxidant activity was obtained as previously reported [3]. In fact, the method was based on the ability of tested samples to block lipoxygenase activity by measuring the absorbance of the resulting solutions at 234 nm (see Section 2.5). As it can be observed from Figure 13, the inhibitory capacity of the samples depends on their concentration but always keeps the following series: DA:βCD > DA > βCD. 

Moreover, the DA:βCD inclusion complex of 1:1 molar ratio begins to manifest inhibitory capacity against lipoxygenase at a small concentration (approximately 0.4 µg/mL concentration in the final solution) which increases with the increasing DA concentration, administered as its inclusion complex, reaching 56% in its inhibitory capacity at 3.13 DA µg/mL. Meanwhile, parent DA manifests inhibitory capacity at a lower concentration (1.31 µg/mL) but reaches the 55% inhibitory activity at a much higher concentration (20.82 µg/mL), concluding that the DA:βCD (1:1) inclusion complex showed better activity compared to the parent DA. This is also confirmed by the IC50 values. Thus, for the parent substance, a concentration of 16.53 ± 0.24 μg/mL, and for the inclusion compound a concentration of 1.93 ± 0.05 μg/mL, are required to achieve a 50% inhibition ability. The stronger inhibitory effect of the inclusion complex can be explained by its larger size compared to the parent DA, blocking the access of linoleic acid to the enzyme center, leading to inhibiting lipoxygenase activity [40]. 

#### 3.3.2. Evaluation of the Drug Release Profile in Blood Plasma from Treated Rats

**Figure 14:**
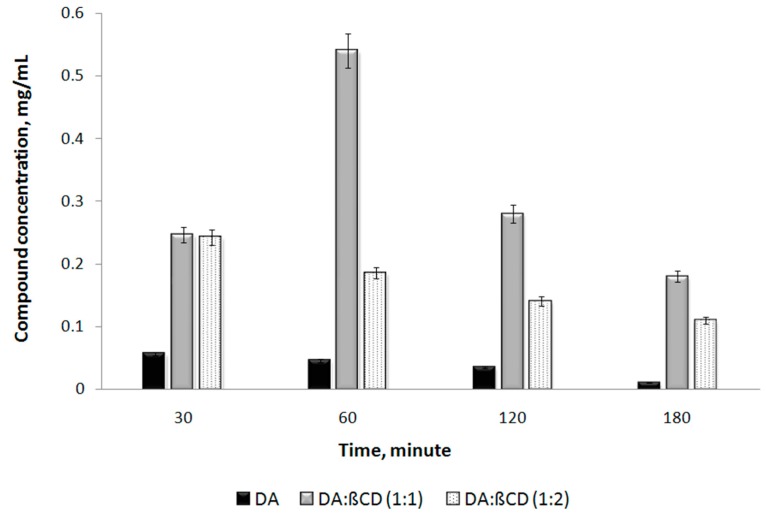
DA concentration in rats’ plasma as a function of time and types of administered compound.

The aim of this study was to comparatively assess the bioavailability of DA and its complex with beta-cyclodextrin of 1:1 and 1:2 molar ratios after a single dose administration in Wistar rats (see Section 2.6). The DA:βCD in a molar ratio of 1:2 was used for comparing native DA with the DA:βCD complex of 1:1 molar ratio. The blood samples were collected and prepared for HPLC analyses following the protocol described in Section 2.6 from Materials and Methods. From all obtained chromatograms, the DA-specific picks areas were determined. By using a DA calibration curve (Figure 11), the real concentrations of DA from blood plasma samples were obtained, which led to determining the variation of DA concentration as a function of sampling time and types of the administered compound in Wistar rats (Figure 14). Herein, we can observe that the DA concentration was always much higher in the blood plasma of rats when the DA:βCD inclusion complex of 1:1 molar ratio was administered (i.e., at 60 min, DA was 11 times higher when the DA:βCD inclusion complex was administered compared to the parent DA one). This confirms our request to increase DA bioavailability, protecting the DA against its degradation in acidic pH from the stomach and implicitly increasing its amount into the bloodstream, close to the administered dose.

## 4. Conclusions

The formation of the 1:1 molar ratio inclusion complex between DA and βCD was demonstrated by different techniques (in solid state: Raman, DSC, and WAXD, and in solution: ^1^H-RMN and ROESY), its stoichiometry was determined from the Job’s plot data, and the association constant of 608.7 M^−1^ was determined from the Benesi-Hildebrand data.

The IC50 value of the DA:βCD (1:1) inclusion complex against lipoxygenase was 1.93 ± 0.05 μg/mL while that of the parent DA was 16.53 ± 0.24 μg/mL. In vivo tests have shown that the bioavailability of DA has been increased due to its protection against degradation in the acidic pH of the stomach through its encapsulation into βCD’s cavity at amounts close to the administered dose. In contrast, when the parent DA was administered, the determined bioavailability was very low, meaning that a high amount of DA was degraded by the low pH of the stomach. Overall, the obtained data suggest that the administration of DA as its inclusion complex with βCD considerably reduces the necessary administration dose, making it possible to use this formulation in the treatment of diseases that required up to now high doses of parent DA, thus eliminating the risk of administering lethal doses.

## Supplementary Materials

The following are available online at https://www.mdpi.com/1999-4923/11/6/295/s1, Figure S1. (A) The ROESY spectrum of DA:βCD inclusion complex of 1:2 molar ratio, recorded in D_2_O, with suppression of the water signal; (B) Expansion of the ROESY spectrum showing the NOE cross-peaks between diminazene aromatic protons and the internal βCD protons H-3, H-5 and H-6. Figure S2. WAXD diffractograms of DA:βCD (1:1) after 18 h (red diffractogram) and 36 h (green diffractogram). Figure S3. WAXD diffractograms of initial βCD (black-lower); after the freeze-drying process (brown-middle); after 18 hours in water saturated atmosphere (brown-upper). Figure S4. WAXD diffractograms of initial DA (blue-lower); after the freeze-drying process (brown-middle); after 18 hours in water saturated atmosphere (brown-upper). Figure S5. HPLC chromatograms of: (A) DA and (B) DA:βCD (1:1) inclusion complex water solutions of 1 mg/mL DA at pH=1.2. Mobile phase flow: 0.5 mL/min. Figure S6. HPLC chromatograms of water solutions at neutral pH (using PBS) of: (A) DA and (B) DA:βCD (1:1) inclusion complex compounds; mobile phase flow: 1 mL/min.

## Abbreviations

CDs	cyclodextrins
βCD	beta-cyclodextrin
DA	diminazene aceturate
DA:βCD (1:1)	inclusion complex in 1:1 molar ratio of components
DA:βCD (1:2)	lyophilized mixture in 1:2 molar ratio of components
^1^H-NMR	Proton Nuclear Magnetic Resonance
H-H ROESY	Rotating Frame Overhause Effect
DSC	Differential Scanning Calorimetry
HPLC	High Performance Liquid Chromatography
WAXD	Wide angle X-ray Diffraction

## References

[1] Garg R. (2012). Supramolecular Chemistry of Host-Guest Inclusion Complexes.

[2] Corciova A., Ciobanu C., Poiata A., Nicolescu A., Drobota M., Varganici C.D., Pinteala T., Fifere A., Marangoci N., Mircea C. (2014). Inclusion complexes of hesperidin with hydroxypropyl-β-cyclodextrin. Physico-chemical characterization and biological assessment. Dig. J. Nanomater. Bios..

[3] Corciova A., Ciobanu C., Poiata A., Mircea C., Nicolescu A., Drobota M., Varganici C.D., Pinteala T., Marangoci N. (2015). Antibacterial and antioxidant properties of hesperidin: Beta-cyclodextrin complexes obtained by different techniques. J. Incl. Phenom. Macrocycl. Chem..

[4] Han Y., Liu W., Huang J., Qiu S., Zhong H., Liu D., Liu J. (2018). Cyclodextrin-based metal-organic frameworks (CD-MOFs) in pharmaceutics and biomedicine. Pharmaceutics.

[5] Marangoci N., Mares M., Silion M., Fifere A., Varganici C., Nicolescu A., Deleanu C., Coroaba A., Pinteala M., Simionescu B.C. (2011). Inclusion complex of a new propiconazole derivative with β-cyclodextrin: NMR, ESI–MS and preliminary pharmacological studies. Res. Pharm. Sci..

[6] Chen G., Jiang M. (2011). Cyclodextrin-based inclusion complexation bridging supramolecular chemistry and macromolecular self-assembly. Chem. Soc. Rev..

[7] Upadhyay A.J., Singh S., Chhipa R.R., Vijayakumar M.V., Ajay A.K., Bhat M.K. (2006). Methyl-β-cyclodextrin enhances the susceptibility of human breast cancer cells to carboplatin and 5-fluorouracil: Involvement of Akt, NF-κB and Bcl-2. Toxicol. Appl. Pharmacol..

[8] Zarzycki P.K., Fenert B., Głód B.K., Grumezescu A.M. (2016). Cyclodextrins-based nanocomplexes for encapsulation of bioactive compounds in food, cosmetics, and pharmaceutical products: Principles of supramolecular complexes formation, their influence on the antioxidative properties of target chemicals, and recent advances in selected industrial applications. Encapsulations, Nanotechnology in the Agri-Food Industry.

[9] Kuriakose S., Uzonna J.E. (2014). Diminazene aceturate (Berenil), a new use for an old compound?. Int. Immunopharmacol..

[10] da Silva Oliveira G.L., de Freitas R.M. (2015). Diminazene aceturate—An antiparasitic drug of antiquity: Advances in pharmacology & therapeutics. Pharmacol. Res..

[11] Dhawale V.S., Amara V.R., Karpe P.A., Malek V., Patel D., Tikoo K. (2016). Activation of angiotensin-converting enzyme 2 (ACE2) attenuates allergic airway inflammation in rat asthma model. Toxicol. Appl. Pharmacol..

[12] Souza L.K., Nicolau L.A., Sousa N.A., Araujo T.S., Sousa F.B., Costa D.S., Souza F.M., Pacifico D.M., Martins C.S., Silva R.O. (2016). Diminazene aceturate, an angiotensin-converting enzyme II activator, prevents gastric mucosal damage in mice: Role of the angiotensin-(1–7)/mas receptor axis. Biochem. Pharmacol..

[13] Bennion D.M., Haltigan E.A., Irwin A.J., Donnangelo L.L., Regenhardt R.W., Pioquinto D.J., Purich D.L., Sumners C. (2015). Activation of the neuroprotective angiotensin-converting enzyme 2 in rat ischemic stroke. Hypertension.

[14] Qiu Y., Shil P.K., Zhu P., Yang H., Verma A., Lei B., Li Q. (2014). Angiotensin-converting enzyme 2 (ACE2) activator diminazene aceturate ameliorates endotoxin-induced uveitis in mice. Investig. Ophthalmol. Vis. Sci..

[15] Foureaux G., Nogueira J.C., Nogueira B.S., Fulgêncio G.O., Menezes G.B., Fernandes S.O.A., Cardoso V.N., Fernandes R.S., Oliveira G.P., Franca J.R. (2013). Antiglaucomatous effects of the activation of intrinsic angiotensin-converting enzyme 2. Investig. Ophthalmol. Vis. Sci..

[16] Neidhart M., Karouzakis E., Jüngel A., Gay R.E., Gay S. (2014). Inhibition of spermidine/spermine N1-acetyltransferase activity: A new therapeutic concept in rheumatoid arthritis. Arthritis Rheumatol..

[17] Jin X., Macdonald D., Staunton J., Wilson A.B. (2008). Methods and Compositions for the Treatment of Neurodegenerative. Disorders. Patent.

[18] Coma M., Aloy P., Pujol A., Gomis X., Oliva B., Lleó A., Mas J.M. (2013). New Combination Therapies for Treating Neurological. Disorders. Patent.

[19] Ge P., Yao X., Li J., Jiang R., Dai J., Zhang L. (2018). Diminazene aceturate alleviated lipopolysaccharide/D-galactosamine induced fulminant hepatitis in mice. Biomed. Pharmacother..

[20] Miller D.M., Swan G.E., Lobetti R.G., Jacobson L.S. (2005). The pharmacokinetics of diminazene aceturate after intramuscular administration in healthy dogs. J. S. Afr. Vet. Assoc..

[21] Tiwari G., Tiwari R., Rai A.K. (2010). Cyclodextrins in delivery systems: Applications. J. Pharm. Bioallied Sci..

[22] Akode R.M., Shantier S.W., Gadkariem E.A., Mohamed M.A. (2017). Simultaneous determination and stability studies on diminazene diaceturate and phenazone using developed derivative spectrophotometric method. Int. J. Anal. Chem..

[23] Lupu A.-C., Barbacariu A., Roman C., Mîndru R., Martinescu G.-V., Cîmpan A.-A., Miron L.D. (2018). In vivo study of conjugated diminazene aceturate for ichthyophthiriosis of farmed carp. Lucrări Științifice Medicină Veterinară.

[24] Spulber M., Pinteala M., Harabagiu V., Simionescu B.C. (2008). Inclusion complexes of sulconazole with beta-cyclodextrin and hydroxypropyl beta-cyclodextrin: Characterization in aqueous solution and in solid state. J. Incl. Phenom. Macrocycl. Chem..

[25] Lewis L.N., Sumpter C.A., Sprenne E.V., Hedges A.R., Romberger M.L. (1995). Purification of Cyclodextrin Complexes. U.S. Patent.

[26] Fielding L. (2000). Determination of association constants (Ka) from solution NMR data. Tetrahedron.

[27] Malterud K.E., Rydland K.M. (2000). Inhibitors of 15-lipoxygenase from orange peel. J. Agric. Food Chem..

[28] Atsriku C., Watson D.G., Tettey J.N.A., Grant M.H., Skellern G.G. (2002). Determination of diminazene aceturate in pharmaceutical formulations by HPLC and identification of related substances by LC/MS. J. Pharm. Biomed. Anal..

[29] Koshiishi I., Mamura Y., Liu J., Imanari T. (1998). Evaluation of an acidic deproteinization for the measurement of ascorbate and dehydroascorbate in plasma samples. Clin. Chem..

[30] Rossi B., Verrocchio P., Viliani G., Mancini I., Guella G., Rigo E., Scarduelli G., Mariotto G. (2009). Vibrational properties of ibuprofen–cyclodextrin inclusion complexes investigated by Raman scattering and numerical simulation. J. Raman Spectrosc..

[31] Socrates G. (2001). Infrared and Raman Characteristic Group Frequencies: Tables and Charts.

[32] Iliescu T., Baia M., Miclăuş V. (2004). A Raman spectroscopic study of the diclofenac sodium–β-cyclodextrin interaction. Eur. J. Pharm. Sci..

[33] Esme A., Sagdinc S.G. (2013). The vibrational studies and theoretical investigation of structure, electronic and non-linear optical properties of Sudan III [1-{[4-(phenylazo) phenyl]azo}-2-naphthalenol]. J. Mol. Struct..

[34] Zimmermann F., Lippert T., Beyer C., Stebani J., Nuyken O., Wokaun A. (1993). N=N Vibrational frequencies and fragmentation patterns of substituted 1-aryl-3,3-dialkyl-triazenes: Comparison with other high-nitrogen compounds. Appl. Spectrosc..

[35] Mehenni L., Lahiani-Skiba M., Ladam G., Hallouard F., Skiba M. (2018). Preparation and Characterization of spherical amorphous solid dispersion with amphotericin B. Pharmaceutics.

[36] Foureaux G., Franca J.R., Nogueira J.C., Fulgêncio Gde O., Ribeiro T.G., Castilho R.O., Yoshida M.I., Fuscaldi L.L., Fernandes S.O.A., Cardoso V.N. (2015). Ocular inserts for sustained release of the angiotensin–converting enzyme 2 activator, diminazene aceturate, to treat glaucoma in rats. PLoS ONE.

[37] Minea B., Marangoci N., Peptanariu D., Rosca I., Nastasa V., Corciova A., Varganici C.D., Nicolescu A., Fifere A., Neamtu A. (2016). Inclusion complexes of propiconazole nitrate with substituted β-cyclodextrins: The synthesis and in silico and in vitro assessment of their antifungal properties. New J. Chem..

[38] Spulber M., Pinteala M., Fifere A., Harabagiu V., Simionescu B.C. (2008). Inclusion complexes of 5-flucytosine with beta-cyclodextrin and hydroxypropyl-beta-cyclodextrin: Characterization in aqueous solution and in solid state. J. Incl. Phenom. Macrocycl. Chem..

[39] Campbell M., Prankerd R.J., Davie A.S., Charman W.N. (2004). Degradation of berenil (diminazene aceturate) in acidic aqueous solution. J. Pharm. Pharmacol..

[40] Lu W., Zhao X., Xu Z., Dong N., Zou S., Shen X., Huang J. (2013). Development of a new colorimetric assay for lipoxygenase activity. Anal. Biochem..

