# Supplementary material for: β-Cyclodextrin as a Functional Excipient Used for Enhancing the Diminazene Aceturate Bioavailability
Source: Pharmaceutics. 2019 Jun 22;11(6):295. doi: 10.3390/pharmaceutics11060295 (PMC6630424; doi:10.3390/pharmaceutics11060295)
Supplement: Supplementary file 1 [file pharmaceutics-11-00295-s001.pdf]

pharmaceutics

MDPI

Supplementary Materials: β-Cyclodextrin as a Functional Excipient Used for Enhancing the Diminazene Aceturate Bioavailability

Narcisa Marangoci, Daniel Timpu, Andreia Corciova, Cornelia Mircea, Anca-Roxana Petrovici, Alina Nicolescu, Elena-Laura Ursu, Valentin Nastasa, Andra-Cristina Bostanaru, Mihai Mares, Mihaela Pertea and Mariana Pinteala

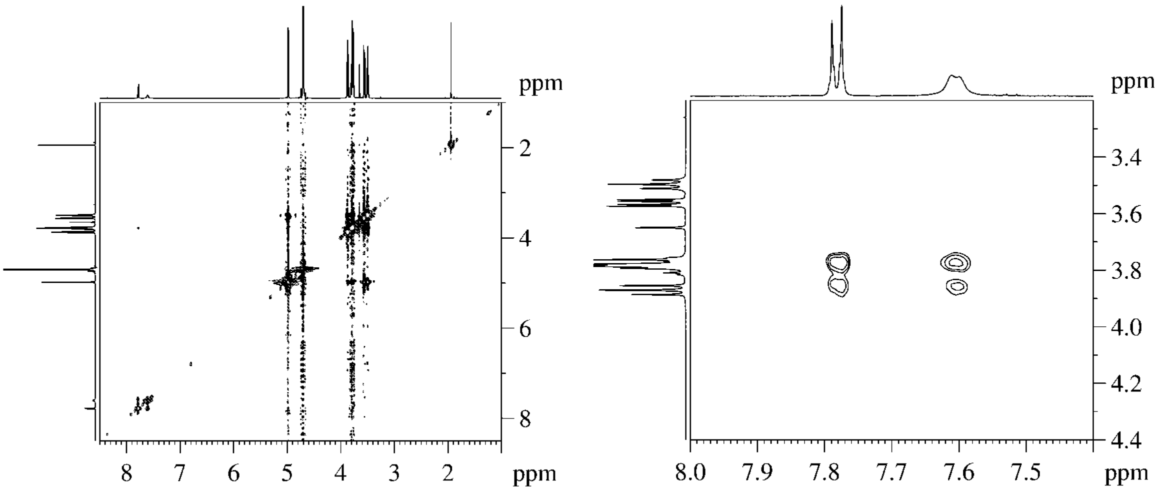

(A) (B)

Figure S1. (A) The ROESY spectrum of DA:βCD inclusion complex of 1:2 molar ratio, recorded in D_2O, with suppression of the water signal; **(B)** Expansion of the ROESY spectrum showing the NOE cross-peaks between diminazene aromatic protons and the internal βCD protons H-3, H-5 and H-6.

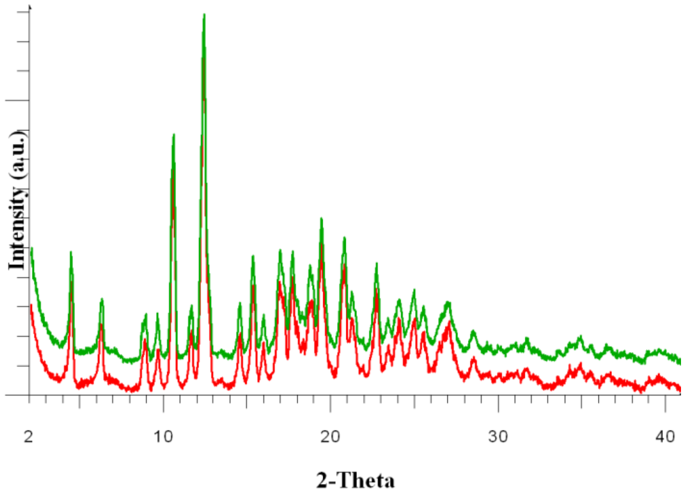

Figure S2. WAXD diffractograms of DA:βCD (1:1) after 18 h (red diffractogram) and 36 h (green diffractogram).

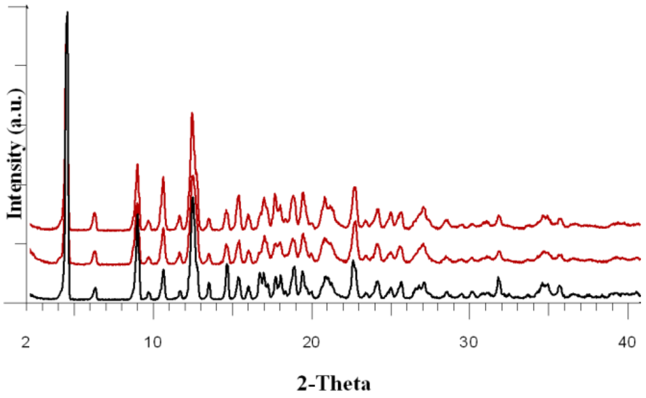

Figure S3. WAXD diffractograms of initial βCD (black-lower); after the freeze-drying process (brown-middle); after 18 hours in water saturated atmosphere (brown-upper).

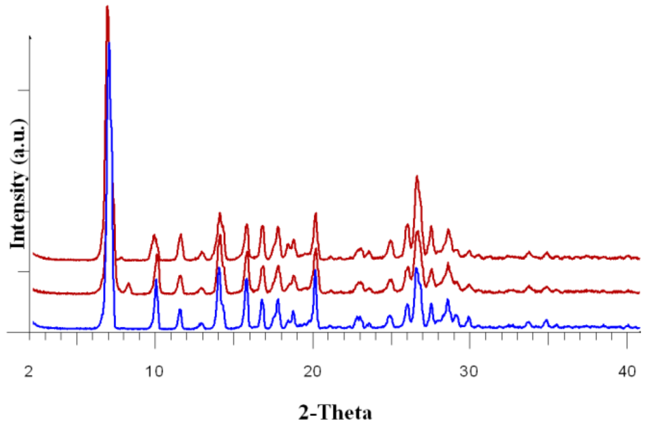

Figure S4. WAXD diffractograms of initial DA (blue-lower); after the freeze-drying process (brown-middle); after 18 hours in water saturated atmosphere (brown-upper).

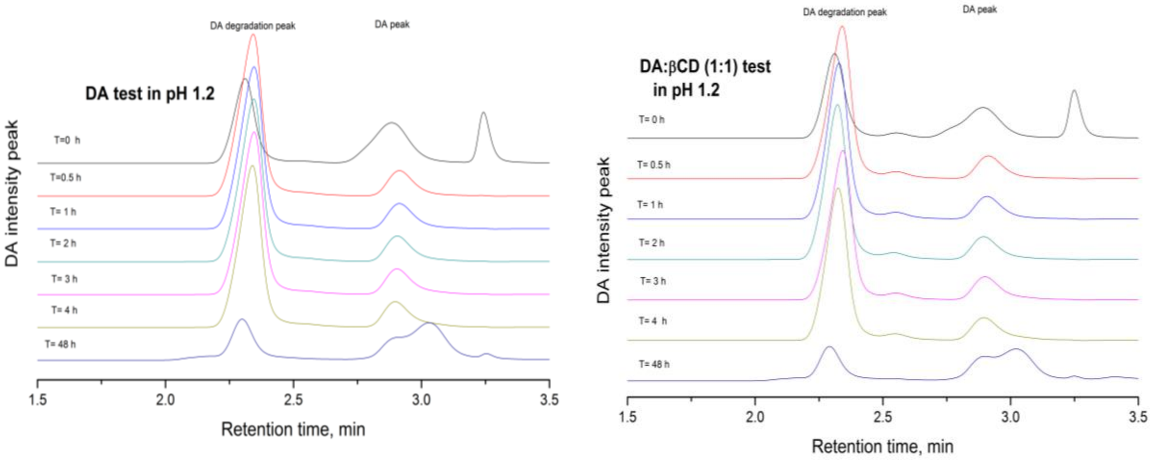

(**A**) (**B**)

Figure S5. HPLC chromatograms of: (**A**) DA and (**B**) DA:βCD (1:1) inclusion complex water solutions of 1 mg/mL DA at pH=1.2. Mobile phase flow: 0.5 mL/min.

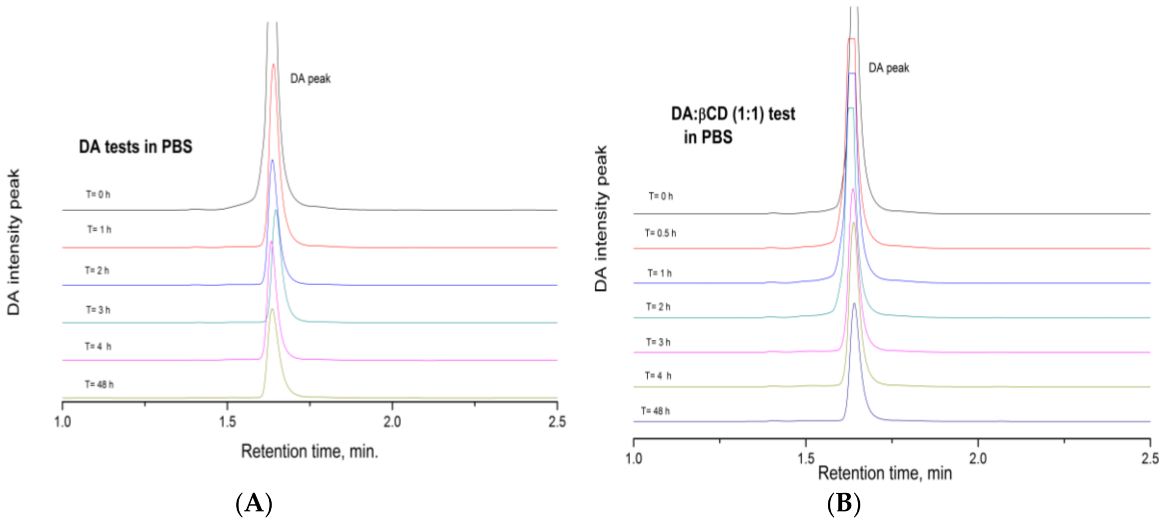

Figure S6. HPLC chromatograms of water solutions at neutral pH (using PBS) of: (**A**) DA and (**B**) DA:βCD (1:1) inclusion complex compounds; mobile phase flow: 1 mL/min.
